# Switching Antipsychotic Medications in People with Schizophrenia: A 4-Year Naturalistic Study

**DOI:** 10.3390/jcm11195965

**Published:** 2022-10-10

**Authors:** Giammarco Cascino, Rossella Ceres, Alessio Maria Monteleone, Paola Bucci, Giulia Maria Giordano, Silvana Galderisi, Palmiero Monteleone

**Affiliations:** 1 Department of Medicine, Surgery and Dentistry, ‘Scuola Medica Salernitana’, University of Salerno, 84131 Salerno, Italy; 2 Department of Psychiatry, University of Campania ‘L. Vanvitelli’, 80138 Naples, Italy

**Keywords:** schizophrenia, antipsychotics, switch, extrapyramidal symptoms, treatment

## Abstract

Although generally effective in ameliorating the core manifestations of schizophrenia, antipsychotics (APs) may lead to only suboptimal responses or may be associated with a variety of treatment-related adverse events which require additional treatment strategies. Under such clinical circumstances, switching APs represents a rational treatment option. The present study aimed to identify the variables that predict AP switch and to quantify the frequency of this phenomenon in people with schizophrenia in real-life. A secondary analysis was conducted on the data collected at baseline and at a 4-year follow-up from a large sample of community-dwelling Italian people with schizophrenia. Demographic and clinical variables as well as information about AP treatment were recorded at two time points. Over the 4-year period, 34.9% of the 571 participants switched the AP; in particular, 8.4% of participants switched from first-generation APs (FGAs) to second-generation APs or vice versa, while 8.2% of them switched to clozapine. Logistic regression models showed that combination of APs at baseline was negatively associated with AP switch, while treatment with FGAs and the presence of extrapyramidal symptoms at baseline were associated with AP class switch. Although the aim of the present study was not to assess predictors of clinical relapse in people with schizophrenia, we might speculate that switching APs represents a surrogate indicator of treatment failure in some patients and could lead into relapse, which is a costly aspect of schizophrenia management in both economic and human terms. The sooner such a negative outcome can be predicted and managed, the sooner the treatment can be optimized to avoid it.

## 1. Introduction

Since 1952, with the development of chlorpromazine, antipsychotic (AP) medications have been mainstays in the treatment of schizophrenia to both manage its acute symptoms and prevent its relapse. Until clozapine was approved by the US Food and Drug Administration for treatment-resistant schizophrenia and second-generation APs (SGAs) were developed, first-generation antipsychotics (FGAs) were widely used as first choice treatment for schizophrenia. Unfortunately, the use of FGAs is associated with side effects, especially extrapyramidal symptoms (EPS) that may increase disease-induced disability and stigma and may require additional treatments [1]. 

The introduction of clozapine and other SGAs broadened treatment options for patients with schizophrenia since these medications have been shown to be as effective as FGAs in relieving the positive symptoms of schizophrenia [2]. Moreover, compared to FGAs, SGAs are associated with a different side effect profile, characterized by weight gain and disturbances in glucose and lipid metabolism [3,4,5,6] as well as other emerging effects (e.g., [7]) but with a less frequent occurrence of EPS [8].

Although generally effective in ameliorating the core manifestations of the disease, both FGAs and SGAs could lead to only suboptimal responses or may be associated with a variety of treatment-related adverse events which require additional treatment strategies. Partial response with persistent positive and negative symptoms and/or the presence of residual symptoms is common even in first-episode patients [9], and relapses frequently occur [10]. Under such clinical circumstances, a switch of APs represents a rational treatment option, in the hope that it results in better treatment outcomes, even if a recent meta-analysis showed that continuing AP treatment at standard doses or switching to a different AP are similarly effective strategies to prevent relapses [11]. Similarly, both life-threatening and other non-dangerous adverse effects, which could shorten the patient’s life expectancy [12] or may impair the patient’s adherence to treatment, could be a reason for changing APs after a benefit/tolerability profile with the patient has been established [13]. 

Few studies have investigated so far the frequency of AP switching and the predictors of APs changes in naturalistic clinical settings. Weinmann et al. [14] evaluated switching from FGAs to SGAs among inpatients with schizophrenia, which is not representative of patients treated in usual outpatient care settings. They found that patients who switched from FGAs to SGAs had fewer previous psychiatric admissions, a shorter illness duration, and fewer comorbid substance disorders. Sernyak et al. [15] used the Veterans Affairs national administrative data to identify predictors of medication switching among patients with schizophrenia. After controlling for independent sociodemographic, diagnostic, and functional variables, the frequency of clinical contact was the most robust predictor of AP switch. Finally, Nyhuis et al. [16] conducted a post hoc analysis of data from a one-year randomized, open-label, multisite study of APs in the treatment of schizophrenia. They found that about one-third of patients switched APs before the end of the study, and that lack of antipsychotic use in the prior year, pre-existing depression, female gender, worsening of AP-induced akathisia, and worsening of symptoms of depression/anxiety during the first 2 weeks of AP therapy were the best predictors of AP switch.

These results cannot be considered conclusive since those studies have several methodological limitations, such as the relatively low number of investigated variables, the poor representativeness of study populations and of outpatient clinical practice settings, and the relatively short follow-up period. Since the identification of treatment switching predictors can help the clinician to tailor effective treatment regimens to patients and optimize early treatment responses, further studies aiming to identify the variables that predict AP switch and the frequency of this phenomenon in people with schizophrenia in the real-life are warranted. To accomplish these aims, a secondary analysis was conducted on the data collected from a large and well-characterized sample of community-dwelling Italian people with schizophrenia, recruited in the context of a multicenter study of the Italian Network for Research on Psychoses (NIRP) [17,18]. In that study sociodemographic and illness-related variables, personal resources and context-related factors that could affect the functional outcome of people with schizophrenia in real-life were recorded. Based on the above literature studies focusing on the possible role of clinical factors in predicting AP switch, we included in the present analysis only the available sociodemographic and illness-related variables. 

## 2. Materials and Methods

### 2.1. Participants

The NIRP conducted a large multicenter study (baseline study) involving 921 community-dwelling, clinically stable patients with schizophrenia, aiming to investigate illness-related variables, personal resources, and context-related factors that could affect the social functioning of people with schizophrenia in the real-life [17]. After 4 years from the baseline study, all the 921 patients were asked to participate in a follow-up study aiming to investigate the natural evolutions of the patterns of relationships among illness-related variables, personal resources, context-related factors and real-life functioning [18]. The inclusion criterion was a diagnosis of schizophrenia according to DSM-IV, confirmed by the Structured Clinical Interview for DSM-IV-Patient version (SCID-I-P). Exclusion criteria were: (1) a history of head trauma with loss of consciousness in the 4-year interval between baseline and follow-up; (2) progressive cognitive deterioration possibly due to dementia or other neurological illness diagnosed in the last 4 years; (3) alcohol and/ or substance abuse in the last 6 months according to the SCID-I-P; (4) current pregnancy or lactation; (5) inability to provide an informed consent; (6) treatment modifications and/or hospitalization due to symptom exacerbation in the last 3 months in order to assess patients who were in a stable state of the disorder.

After receiving a comprehensive explanation of the study procedures and goals, all the subjects signed a written informed consent to participate. All the study procedures complied with the ethical standards of the relevant national and institutional committees on human experimentation and with the Helsinki Declaration of 1975, as revised in 2008. The study was approved by the Ethics Committees of participating centers. Recruitment took place from March 2016 to December 2017.

### 2.2. Clinical Assessment

Positive and disorganization symptoms were assessed by the Positive and Negative Syndrome Scale (PANSS) [19]. Scores for “positive symptoms” were calculated based on the 7 consensus, 5-factor solution proposed by Wallwork et al. [20]. “Disorganization” was the PANSS item P2, to avoid overlap with cognitive impairment. Since the PANSS is not considered an adequate instrument for the assessment of avolition, does not assess anhedonia, and the evaluation of asociality overlaps with measures of functioning [21,22,23,24,25], negative symptoms were measured by means of the Brief Negative Symptom Scale (BNSS) [26] that allows the identification of two separate factors: (a) avolition, consisting of anhedonia, asociality, and avolition and (b) expressive deficit, including blunted affect and alogia. The Italian version of the BNSS was validated as part of the Italian Network project [27]. Depressive symptoms were evaluated using the Calgary Depression Scale for Schizophrenia (CDSS) [28]. EPS were assessed by the St. Hans Rating Scale (SHRS), which allowed us to assess dyskinesia, parkinsonism, dystonia, and akathisia [29].

All included subjects had a stable drug treatment dose and were on the same AP drug/s within the 3 months before both the baseline and the follow-up assessment. 

Patients were treated with different FGAs and/or SGAs according to judgment of the referring clinicians. For each patient, APs taken at baseline and at follow-up were recorded. Patients who took at follow-up an AP different from that taken at baseline were considered switchers. This categorization was carried out first for any change in APs, then for a switch from FGAs to SGAs or vice versa and finally for a switch to clozapine. Daily chlorpromazine equivalent doses (CED) of APs were calculated as suggested by Gardner et al. [30].

### 2.3. Statistical Analysis

All statistical analyses were performed through R, Version 4.2 (R core Team, Vienna, Austria).

Multivariate analyses of variance (MANOVAs) were run to investigate differences in demographic and clinical variables between participants who switched APs and those who did not according to the above categorization. Group differences in categorical variables were investigated by the Pearson’s chi square test.

Different logistic regression models were performed to identify factors associated with AP switch: dependent variable was the first switch for any APs, then a switch between APs generation, and last a switch to clozapine, while patient age, gender, duration of illness, psychopathology severity, presence of any EPS, class and combination of APs taken at baseline, long-acting AP formulation, and the CED of AP taken at follow-up were included as independent variables. Class of AP taken at baseline was categorized as FGA or SGA, in case of a combination of an FGA with an SGA, FGA was assigned; combination of AP was a dummy variable in which any combination of AP, of both same and different class, was indicated with ‘1′; the long-acting AP formulation was also a dummy variable. 

Finally, to obtain the magnitude of the variation on the probability scale, marginal effects at the mean, in which the covariates are kept at their mean values, were used.

## 3. Results

Of the 921 subjects recruited at baseline, 618 joined the follow-up study. No significant differences in baseline variables emerged between participants joining the follow-up study and those who did not (*n* = 303) [18]. Complete assessments were available for 571 participants who were included in this analysis. They were 391 men (mean age ± SD: 44.6 ± 10.1) and 180 women (mean age ± SD: 46.1 ± 11.1). At baseline, 406 patients were treated with SGAs (339 with single SGA, 67 with two or more different SGAs), 85 were treated with FGAs (66 with single FGA, 89 with two or more FGAs), and 80 with a combination of FGAs and SGAs. At the follow-up assessment, 413 participants were treated with SGAs (341 with single SGA, 72 with two or more different SGAs), 77 with FGAs (71 with single FGA, 6 with two or more FGAs), and 81 with a combination of FGAs and SGAs, respectively. At baseline, 85 patients were treated with long-acting AP, while at follow-up, the number treated with long-acting AP was 135. At baseline, the mean (±SD) daily CED of APs was 516.45 ± 347.01 mg, while at follow-up it was 353.13 ± 273.28 mg.

Over the 4-year period, 199 (34.9%) participants switched AP; 105 (18.4%) participants switched the class of AP. In particular, 49 (8.6%) participants switched from FGAs to SGAs, while 56 (9.8%) patients had the opposite switch; finally, 47 (8.2%) patients switched to clozapine. 

Demographic and clinical characteristics of participants at baseline according to the AP switch are reported in Table 1. Number and percentages of patients who switched the baseline AP at the study end point according to the medication at baseline are reported in Supplementary Table S1.

At baseline assessment, the MANOVA on demographic and clinical measures comparing the group who switched the AP with the group who did not switch did not show a significant overall group effect (Pillai trace = 0.01, F_8, 406_ = 0.48, *p* = 0.87). The MANOVA comparing the group who switched from FGA to SGA or vice versa and the group who did not switch AP class did not show a significant group effect (Pillai trace = 0.01, F_8, 406_ = 0.57, *p* = 0.8). Finally, the MANOVA comparing the group who switched to clozapine with the group who did not switch to clozapine showed a significant overall group effect (Pillai trace = 0.04, F_8, 406_ = 2.09, *p* = 0.03). Indeed, a significant difference emerged between the two groups in age (F_1, 413_ = 10.2, *p* = 0.001), since the group who switched to clozapine over the 4-year period was younger (35.81 ± 10.68 years) than the group who did not switch (40.85 ± 10.35 years).

The Pearson’s chi square test showed a significant association between the switch of AP class and the AP class taken at baseline (χ^2^ = 37.4, *p* = 9.7 × 10^−10^) and the presence of any EPS at baseline (χ^2^ = 13.2, *p* = 2.7 × 10^−4^), while no significant association emerged between the switch of AP class and gender as well as between the switch to any AP or to clozapine and all the categorical variables. 

**Figure 1:**
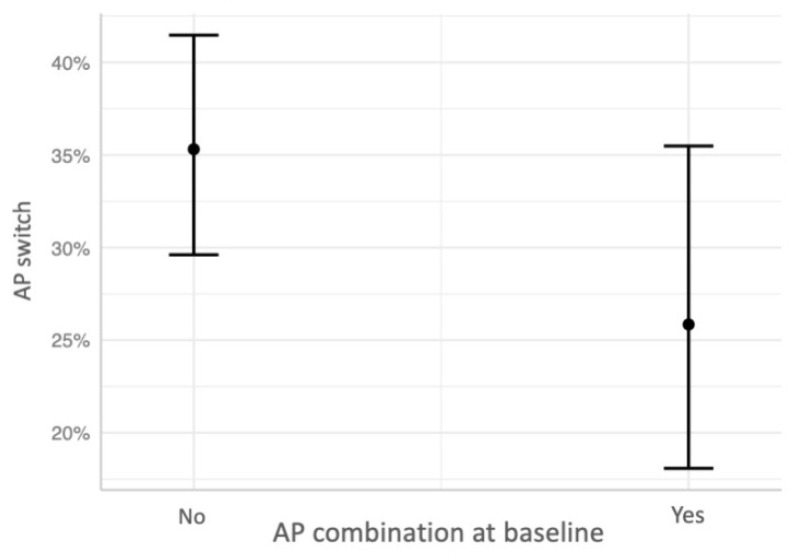
Predicted probabilities of antipsychotic (AP) switch associated with APs combination at baseline.

**Figure 2:**
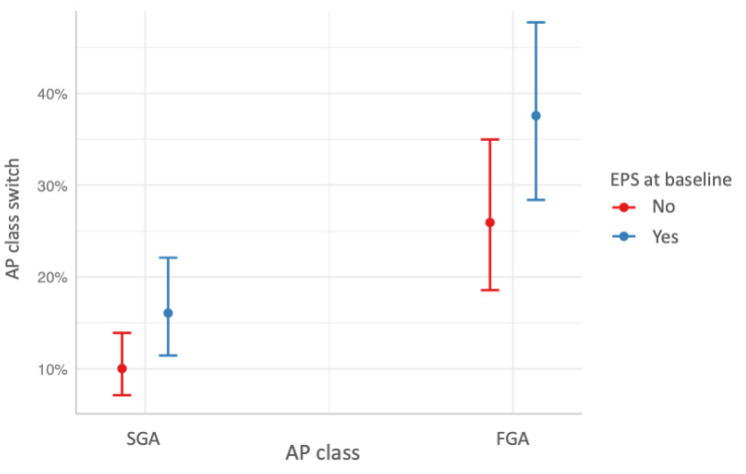
Predicted probabilities of switching antipsychotic (AP) class associated with AP class taken at baseline and the occurrence of extrapyramidal symptoms (EPS) at baseline. FGA = first-generation antipsychotics; SGA = second-generation antipsychotics.

The first logistic regression model with any AP switch as dependent variable showed that any AP switch was negatively associated with the combination of APs at baseline (b = −0.44, *p* = 0.04), indicating that participants taking more than one AP at baseline were less likely to switch APs over the follow-up period (Figure 1). The logistic regression model with AP class switch as dependent variable showed that treatment with FGAs (b = 1.09, *p* = 3.75 × 10^−5^) and the presence of any EPS (b = 0.52, *p* = 0.02) at baseline were significantly associated to AP class switch, indicating that participants taking FGA and showing EPS at baseline were more likely to switch AP class over the follow-up period (Figure 2). Finally, the logistic regression model with switch to clozapine as dependent variable showed that age was negatively associated to clozapine switch (b = −0.08, *p* = 0.01), while the severity of positive symptoms was positively associated to clozapine switch (b = 0.1, *p* = 0.04), indicating that younger participants with more severe positive symptoms at baseline were more likely to switch to clozapine over the follow-up period.

## 4. Discussion

The first finding of the present secondary analysis of data from the naturalistic multicentre study of the NIRP was that approximately one-third (34.9%) of the participants switched AP over the 4-year follow-up period. This finding is in line with two previous studies showing that at 1-year follow-up, AP switch occurred in 26.3% and 29.5% of participants, respectively [15,16]. Different from these results, the CATIE study reported that 74% of patients discontinued AP medication within 18 months from starting treatment and switched to a different AP in phase II of the study. In addition to the clear methodological differences between the CATIE study and the present one, it has been suggested that such a high rate of switching may be due to the CATIE study protocol, which encouraged patients and clinicians to switch drugs too soon with the hope that a new drug might produce better results than that originally assigned [31], although subsequent analyses on the CATIE trial data showed that switching to a new medication yielded no advantage over staying on the previous medication [32].

We found that none of the variables assessed at baseline predicted the switch to any AP at follow-up except for the presence of multiple AP therapy. Indeed, participants who took more than one AP at baseline were less likely to switch AP medication over the 4-year naturalistic treatment. The discrepancy in our findings with those of the previous studies [15,16] may be due, at least in part, to differences in the variables entered in the regression model and/or to the longer follow-up period of our study, which could have an impact on the effect of some clinical variables. The finding of a negative association between combination of APs at baseline and AP switch over the follow-up suggests that the combination of APs is considered by clinician an alternative to AP switch in case of an unsatisfactory response despite most of the evidence and treatment guidelines recommending AP monotherapy and acknowledging the feasibility of AP combination only in specific conditions, such as for clozapine-resistant patients [33,34,35,36,37]. In support of this possible explanation, at baseline participants with AP polytherapy showed positive and disorganization symptoms higher than those with AP monotherapy (post hoc analysis: t = 4.4, *p* < 0.001; t = 2.85; *p* = 0.005, respectively). 

Our second study finding was that 18.4% of the total sample switched AP class with 8.6% of participants switching from FGAs to SGAs and 9.8% of them having the opposite switch. Inconsistent with our data, the CUtLASS study showed that 35% of patients switched from an SGA to an FGA, while 46% had the opposite switching within 1 year from the treatment randomization [38]. Differences in the study designs and the length of the follow-up periods may be the major determinants of such a discrepancy. Moreover, we found that treatment with FGA and the presence of EPS at baseline were significantly associated to the AP class switch. The latter associations were expected since FGAs are associated with a prevalence of EPS higher than SGA and the occurrence of EPS may be considered a valid reason to switch from FGAs to SGAs since EPS increase disease-induced disability and stigma, impair the patients’ adherence to treatment, and may require additional treatments, which, in turn, may impair the drug treatment tolerability [8,39]. Consistent with our findings, Nyhuis et al. [16] found that worsening of akathisia in the first 2 weeks of AP treatment predicted AP switch in a sample composed almost completely of outpatients from a randomized open-label study conducted in a naturalistic setting. On the contrary, Weinmann et al. [14] found that a short disease duration, fewer previous psychiatric hospitalizations, voluntary admission, and pronounced thought disorder were significantly associated with switching from FGAs to SGAs. This discrepancy may be due to the different clinical setting and the different follow-up duration, as Weissman et al. [14] focused on inpatients with 1-year follow-up. 

Our last study finding was that 8.2% of participants switched to clozapine. Younger age and more severe positive symptoms were associated with clozapine switch. These findings are in line with clinical recommendations to offer clozapine monotherapy as soon as the criteria for treatment resistance are fulfilled [37]. 

Some limitations of the present study need to be acknowledged. First, detailed information about drug treatments over the 4-year follow-up is lacking, so we were not able to establish when the switch occurred over follow-up or to identify the reasons for switching or to verify whether patients underwent multiple AP switches. Second, the patients’ adherence to treatment was not assessed and this may have affected the switch rate since clinicians could have switched the AP in the presence of an apparent non-clinical response to the ongoing treatment. Moreover, anamnestic information about substance abuse may be quite unreliable and nicotine abuse, which significantly affects the pharmacokinetics of clozapine [40], was not assessed. Information regarding psychopharmacological treatments other than AP, in particular antidepressants, was lacking. In order to overcome this issue, since depressive symptoms were found to be the best predictors of AP switch [16], we included CDSS, as a measure of depressive symptoms, in the analysis. Finally, side effects of AP, such as the increase in weight or diabetes, were not reported, although they may be a reason to switch AP. 

The strengths of our study include its naturalistic nature, the large sample of participants who were recruited in specialist mental health services distributed throughout the whole national territory, and the long follow-up duration. 

## 5. Conclusions

In conclusion, the present findings, showing that approximately one-third of the participants switched AP over the 4-year follow-up period and that combination of APs was negatively associated with AP switch, while treatment with an FGA and the presence of EPS at baseline were associated to the AP class switch, might help inform clinical decision-making in usual practice. The current management of patients with primary psychosis worldwide is often stereotyped, since in almost all cases an AP is prescribed, with SGA usually preferred to FGA [41,42,43]. Tailoring effective treatment regimens to patients and optimizing early treatment responses are pivotal challenges in psychiatry [44,45,46,47]. Although the aim of the present study was not to assess predictors of clinical relapse in people with schizophrenia living in the community, we might speculate that switching APs represents a surrogate indicator of treatment failure in some patients. As treatment failure often leads into relapse, which is one of the costliest aspects of schizophrenia management in both economic and human terms [48,49], the sooner such a negative outcome can be predicted and managed, the sooner the treatment can be optimized to avoid it. 

## Figures and Tables

**Table 1:** Demographic and clinical characteristics of study participants at baseline according to the occurrence of antipsychotic switch. AP, antipsychotics; CDSS, Calgary Depression Scale for Schizophrenia; CED, chlorpromazine equivalent doses; EPS, extrapyramidal symptoms; FGA, first-generation antipsychotics; SGA, second-generation antipsychotics.

	No AP Switch (*n* = 372)	AP Switch		
		Total Group (*n* = 199)	AP Class Switch (*n* = 105)	Clozapine Switch (*n* = 47)
Age, years	40.6 ± 10.1	40.1 ± 11.1	42.8 ± 9.5	35.8 ± 10.6 *
Gender, m (%)	248 (66.7)	143 (71.8)	77 (73.3)	33 (70.2)
Illness duration, yrs	16.4 ± 10.1	16.6 ± 10.7	19.4 ± 10.4	13.8 ± 9.5
AP generation, FGA (%)	110 (29.6)	55 (27.6)	56 (53.3)	14 (29.8)
SGA (%)	262 (70.4)	144 (72.4)	49 (46.7)	33 (70.2)
AP combination, *n* (%)	118 (31.7)	48 (24.1)	45 (42.8)	12 (25.5)
AP long-acting, *n* (%)	50 (13.4)	35 (17.6)	15 (14.3)	7 (14.9)
Daily CED	516.6 ± 350.1	361.2 ± 302.1	424.5 ± 309.8	549.5 ± 388.7
Positive symptoms	9.7 ± 4.9	9.8 ± 4.4	10.7 ± 5.0	11.1 ± 5.2
Disorganization	2.6 ± 1.5	2.6 ± 1.4	2.9 ± 1.5	2.7 ± 1.5
Expression deficits	12.7 ± 8.3	12.9 ± 7.3	13.7 ± 8.0	13.8 ± 7.4
Avolition	20.7 ± 9.9	21.1 ± 8.9	21.2 ± 8.7	21.3 ± 8.7
CDSS	3.8 ± 3.9	4.1 ± 4.0	4.2 ± 3.8	4.4 ± 4.4
Any EPS, *n* (%)	145 (39)	86 (43.2)	59 (56.2)	19 (40.4)

* *p* = 0.001 vs. no AP switch.

## Data Availability

The data presented in this study are available on request from the corresponding author.

## Supplementary Materials

The following supporting information can be downloaded at: https://www.mdpi.com/article/10.3390/jcm11195965/s1. Table S1. Number and percentages of patients who switched the baseline antipsychotic (AP) at the study end point according to the medication at baseline.
