# Supplementary material for: Switching Antipsychotic Medications in People with Schizophrenia: A 4-Year Naturalistic Study
Source: J Clin Med. 2022 Oct 10;11(19):5965. doi: 10.3390/jcm11195965 (PMC9573332; doi:10.3390/jcm11195965)
Supplement: Supplementary file 1 [file jcm-11-05965-s001.zip › jcm-1931069-supplementary.pdf]

Supplementary Table S1. Number and percentages of patients who switched the baseline antipsychotic (AP) at the study end point according to the medication at baseline

AP Class	AP combination	Long-acting		No switch	AP switch	AP class switch	Swtich to Clozapine	Total
SGA	No	No	n	199	63	23	26	311
			%	64	20	7.4	8.4	
		Yes	n	14	10	2	2	28
			%	50	35.8	7.1	7.1	
	Yes	No	n	40	4	4	4	52
			%	76.9	7.7	7.7	7.7	
		Yes	n	9	3	2	1	15
			%	60	20	13.3	6.7	
		Total	n	262	80	31	33	406
			%	64.5	19.7	7.6	8.1	
FGA	No	No	n	30	4	10	7	51
			%	58.8	7.8	19.6	13.7	
		Yes	n	11	1	3	0	15
			%	73.3	6.7	20.0		
	Yes	No	n	53	10	6	3	72
			%	73.6	13.9	8.3	4.2	
		Yes	n	16	5	2	4	27
			%	59.3	18.5	7.4	14.8	
		Total	n	110	20	21	14	165
			%	66.7	12.1	12.7	8.5	
